# Supplementary material for: Basal ganglia output (entopeduncular nucleus) coding of contextual kinematics and reward in the freely moving mouse
Source: eLife. 2025 Feb 26;13:RP98159. doi: 10.7554/eLife.98159 (PMC11864757; doi:10.7554/eLife.98159)
Supplement: Supplementary file 1. — Details of the statistics used for each figure. [file elife-98159-supp1.docx]

Table 1. Statistical table

| Figure | Descriptive statistics | Type of test | p value | n |
| --- | --- | --- | --- | --- |
| 1E | Mean +/- SEM  Simple linear regression | Correlation coefficient two-tailed permutation  test | 0.006 | 7 data points |
| 1H | Mean +/- SEM  Simple linear regression | Correlation coefficient two-tailed permutation  test | Correct trials=0.004  Incorrect trials= 0.17 | 7 data points |
| 1J | Mean +/- SEM  Simple linear regression | Correlation coefficient two-tailed permutation  test | Correct trials=0.30  Incorrect trials= 0.097 | 7 data points |
| 2I | Mean zScore | Wilcoxon signed-rank test | Return vs Go=0.17  Return vs Wait=0.20  Return vs Contralateral=0.48  Return vs Ipsilateral=0.12  Return vs Correct=4.0x10^-5^  Return vs Incorrect=0.88  Go vs Wait=0.11  Go vs Contralateral=0.43  Go vs Ipsilateral=0.51  Go vs Correct=0.0011  Go vs Incorrect=0.36  Wait vs Contralateral=0.35  Wait vs Ipsilateral=0.0024  Wait vs Correct=1.2x10^-5^  Wait vs Incorrect=0.42  Contralateral vs Ipsilateral=0.034  Contralateral vs Correct=0.00014  Contralateral vs Incorrect=0.59  Ipsilateral vs Correct=0.0017  Ipsilateral vs Incorrect=0.13  Correct vs Incorrect=1.7x10^-5^ | 118 data points per condition |
| 3-2D | Mean delta R2 (see Methods) | Wilcoxon signed-rank test | **Model comparison**  Stimulus vs Difficulty=1.8x10^-5^  Stimulus vs Interpretation=0.0021  Stimulus vs AngVel=0.012  Difficulty vs Interpretation=0.35  Difficulty vs AngVel=3.9x10^-9^  Interpretation vs AngVel=2.8x10^-8^ | 118 data points per condition |
| 4D | Mean | Wilcoxon signed-rank test | Kine vs spatio=5.1x10^-10^  Kine vs reward=0.016  Kine vs kine+spatio+reward=8.6x10^-20^  Spatio vs reward=0.16  Spatio vs kine+spatio+reward=6.1x10^-20^  Reward vs kine+spatio+reward=1.9x10^-20^ | 118 units |
| 4E | Mean | Wilcoxon signed-rank test | **Kinematic model**  Return vs Go=5.3x10^-8^ | 118 units |
| 4E | Mean | Wilcoxon signed-rank test | **Spatio-temporal model**  Return vs Go=3.2x10^-4^ | 118 units |
| 4E | Mean | Wilcoxon signed-rank test | **Kine+spatio model**  Return vs Go=4.5x10^-6^ | 118 units |
| 4G | Mean +/- SEM | Wilcoxon signed-rank test | **Kinematic model trained on Return data, tested on:**  Return vs Go=2.2x10^-15^  **Kinematic model trained on Go data, tested on:**  Return vs Go=4.5x10^-21^ | 118 units |
| 4G | Mean +/- SEM | Wilcoxon signed-rank test | **Spatio-temporal model trained on Return data, tested on:**  Return vs Go=4.0x10^-19^  **Spatio-temporal model trained on Go data, tested on:**  Return vs Go=5.4x10^-21^ | 118 units |
| 4G | Mean +/- SEM | Wilcoxon signed-rank test | **Kine+spatio model trained on Return data, tested on:**  Return vs Go=2.4x10^-19^  **Kine+spatio model trained on Go data, tested on:**  Return vs Go=4.2x10^-21^ | 118 units |
| 4M | Mean | Mann–Whitney  U test | **Kinematic model**  Rew-pos vs rew-neg=0.015  Rew-pos vs no-rew=0.62  Rew-neg vs no-rew=0.051 | Rew-pos= 51 units  Rew-neg= 22 units  No-rew= 45 units |
| 4M | Mean | Mann–Whitney  U test | **Spatio-temporal model**  Rew-pos vs rew-neg=0.017  Rew-pos vs no-rew=0.79  Rew-neg vs no-rew=0.060 | Rew-pos= 51 units  Rew-neg= 22 units  No-rew= 45 units |
| 4M | Mean | Mann–Whitney  U test | **Kine+spatio model**  Rew-pos vs rew-neg=0.029  Rew-pos vs no-rew=0.61  Rew-neg vs no-rew=0.11 | Rew-pos= 51 units  Rew-neg= 22 untis  No-rew= 45 units |
| 4N | Mean | Wilcoxon signed-rank test | **Kinematic model**  Correct vs incorrect=0.62  Correct vs false alarm= 0.07  Incorrect vs false alarm= 0.19 | 118 units |
| 4N | Mean | Wilcoxon signed-rank test | **Spatio-temporal model**  Correct vs incorrect=0.74  Correct vs false alarm=0.02  Incorrect vs false alarm=0.08 | 118 units |
| 4N | Mean | Wilcoxon signed-rank test | **Kine + spatio model**  Correct vs incorrect=0.72  Correct vs false alarm=0.06  Incorrect vs false alarm=0.12 | 118 units |
| 5E | Pearson correlation coefficient | Pearson correlation coefficient two-tailed permutation test | **Context dPC1 (c-dPC1)**  c-dPC1 vs Head speed=0.54  c-dPC1 vs Body speed=0.96  c-dPC1 vs Head acceleration=0.18  c-dPC1 vs Body acceleration=0.27  c-dPC1 vs angVel=0.88  c-dPC1 vs dist from X=0.0  c-dPC1 vs dist from Y=0.0  c-dPC1 vs dist Waiting=0.0  c-dPC1 vs vel from Waiting corner=0.01  c-dPC1 vs dist Goal=0.62  c-dPC1 vs time=0.46 | 4 time series (1.2s, 100Hz) per dPC vs 4 time series (1.2s, 100Hz) per variable  (480 points vs 480 points) |
| 5H | Pearson correlation coefficient | Pearson correlation coefficient two-tailed permutation test | **Context dPC2 (c-dPC2)**  c-dPC2 vs Head speed=0.53  c-dPC2 vs Body speed=0.57  c-dPC2 vs Head acceleration=0.35  c-dPC2 vs Body acceleration=0.79  c-dPC2 vs angVel=0.77  c-dPC2 vs dist from X=0.0  c-dPC2 vs dist from Y=0.0  c-dPC2 vs dist Waiting=0.0  c-dPC2 vs vel from Waiting corner=0.0  c-dPC2 vs dist Goal=0.57  c-dPC2 vs time=0.85 | 4 time series (1.2s, 100Hz) per dPC vs 4 time series (1.2s, 100Hz) per variable  (480 points vs 480 points) |
| 5-1D | Pearson correlation coefficient | Pearson correlation coefficient two-tailed permutation test | **Temporal dPC1 (t-dPC1)**  t-dPC1 vs Head speed=0.0  t-dPC1 vs Body speed=0.37  t-dPC1 vs Head acceleration=0.0  t-dPC1 vs Body acceleration=0.0  t-dPC1 vs angVel=0.93  t-dPC1 vs dist from X=0.77  t-dPC1 vs dist from Y=0.4  t-dPC1 vs dist Waiting=0.86  t-dPC1 vs vel from Waiting corner=0.32  t-dPC1 vs dist Goal=0.0  t-dPC1 vs time=0.0 | 4 time series (1.2s, 100Hz) per dPC vs 4 time series (1.2s, 100Hz) per variable  (480 points vs 480 points) |
| 5-1E | Pearson correlation coefficient | Pearson correlation coefficient two-tailed permutation test | **Temporal dPC2 (t-dPC2)**  t-dPC2 vs Head speed=0.0  t-dPC2 vs Body speed=0.0  t-dPC2 vs Head acceleration=0.0  t-dPC2 vs Body acceleration=0.05  t-dPC2 vs angVel=0.82  t-dPC2 vs dist from X=0.01  t-dPC2 vs dist from Y=0.05  t-dPC2 vs dist Waiting=0.48  t-dPC2 vs vel from Waiting corner=0.76  t-dPC2 vs dist Goal=0.0  t-dPC2 vs time=0.0 | 4 time series (1.2s, 100Hz) per dPC vs 4 time series (1.2s, 100Hz) per variable  (480 points vs 480 points) |
| 5-1F | Pearson correlation coefficient | Pearson correlation coefficient two-tailed permutation test | **Temporal dPC3 (t-dPC3)**  t-dPC3 vs Head speed=0.32  t-dPC3 vs Body speed=0.77  t-dPC3 vs Head acceleration=0.94  t-dPC3 vs Body acceleration=0.0  t-dPC3 vs angVel=0.67  t-dPC3 vs dist from X=0.7  t-dPC3 vs dist from Y=0.28  t-dPC3 vs dist Waiting=0.65  t-dPC3 vs vel from Waiting corner=0.94  t-dPC3 vs dist Goal=0.0  t-dPC3 vs time=0.0 | 4 time series (1.2s, 100Hz) per dPC vs 4 time series (1.2s, 100Hz) per variable  (480 points vs 480 points) |
| 5-1G | Pearson correlation coefficient | Pearson correlation coefficient two-tailed permutation test | **Side dPC1 (s-dPC1)**  s-dPC1 vs Head speed=0.39  s-dPC1 vs Body speed=0.53  s-dPC1 vs Head acceleration=0.32  s-dPC1 vs Body acceleration=0.18  s-dPC1 vs angVel=0.0  s-dPC1 vs dist from X=0.0  s-dPC1 vs dist from Y=0.0  s-dPC1 vs dist Waiting=0.92  s-dPC1 vs vel from Waiting corner=0.64  s-dPC1 vs dist Goal=0.93  s-dPC1 vs time=0.42 | 4 time series (1.2s, 100Hz) per dPC vs 4 time series (1.2s, 100Hz) per variable  (480 points vs 480 points) |
| 6G, lower | Pearson correlation coefficient | Pearson correlation coefficient two-tailed permutation test | Paw position vs PC1=0.77  Paw position vs PC2=0.81  Paw position vs PC3=0.11  Paw position vs PC4=0.30  Paw velocity vs PC1=0.71  Paw velocity vs PC2=0.52  Paw velocity vs PC3=0.83  Paw velocity vs PC4=0.0 | 200 points vs 200 points |
| 6 I, right | Pearson correlation coefficient | Pearson correlation coefficient two-tailed permutation test | PC4 vs velHead=0.0  PC4 vs velBody=0.0  PC4 vs accHead=0.58  PC4 vs accBody=0.93  PC4 vs angVel=0.07  PC4 vs paw1=0.11  PC4 vs paw2=0.11  PC4 vs paw3=1.0  PC4 vs paw4=0.3  PC4 vs paw1vel=0.0  PC4 vs paw2vel=0.0  PC4 vs paw3vel=0.0  PC4 vs paw4vel=0.0  PC4 vs distX=0.03  PC4 vs distY=0.01  PC4 vs distE=0.05  PC4 vs distGoal=0.0  PC4 vs timeGoal=0.0 | 200 points vs 200 points |
| 6J, left | Mean +/- SEM | Permutation test | **Classifier accuracy, original matrix**  Context original labels vs shuffled=0.0  Step # original labels vs shuffled=0.0  Paw position original labels vs shuffled=0.478  Paw velocity original labels vs shuffled=0.431 | 1 vs 1000 |
| 6J, right | Mean +/- SEM | Permutation test | **Classifier accuracy, PC4-weighted matrix**  Context original labels vs shuffled=0.0  Step # original labels vs shuffled=0.0  Paw position original labels vs shuffled=0.972  Paw velocity original labels vs shuffled=0.0 | 1 vs 1000 |
| 7L | Mean +/- SEM | Permutation test | **Classifier accuracy**  Phase original labels vs shuffled=0.0  Left-right original labels vs shuffled=0.0  Lick # original labels vs shuffled=0.0  Correct vs incorrect original labels vs shuffled=0.0 | 1 vs 1000 |
